# Rapid isothermal amplification and portable detection system for SARS-CoV-2

**DOI:** 10.1073/pnas.2014739117

**Published:** 2020-08-31

**Authors:** Anurup Ganguli, Ariana Mostafa, Jacob Berger, Mehmet Y. Aydin, Fu Sun, Sarah A. Stewart de Ramirez, Enrique Valera, Brian T. Cunningham, William P. King, Rashid Bashir

**Affiliations:** ^a^Department of Bioengineering, University of Illinois at Urbana–Champaign, Urbana, IL 61801;; ^b^Nick Holonyak Jr. Micro and Nanotechnology Laboratory, University of Illinois at Urbana–Champaign, Urbana, IL 61801;; ^c^Department of Mechanical Science and Engineering, University of Illinois at Urbana–Champaign, Urbana, IL 61801;; ^d^Department of Electrical and Computer Engineering, University of Illinois at Urbana–Champaign, Urbana, IL 61801;; ^e^Emergency Medicine, University of Illinois College of Medicine at Peoria & OSF Healthcare, Peoria, IL 61637;; ^f^Carl R. Woese Institute for Genomic Biology, University of Illinois at Urbana–Champaign, Urbana, IL 61801;; ^g^Department of Biomedical and Translational Sciences, Carle Illinois College of Medicine, Urbana, IL 61801

**Keywords:** SARS-CoV-2, COVID-19 diagnostics, point-of-care, smartphone reader, RT-LAMP

## Abstract

The COVID-19 pandemic provides an urgent example where a gap exists between availability of state-of-the-art diagnostics and current needs. As assay protocols and primer sequences become widely known, many laboratories perform diagnostic tests using methods such as RT-PCR or reverse transcription loop mediated isothermal amplification (RT-LAMP). Here, we report an RT-LAMP isothermal assay for the detection of severe acute respiratory syndrome coronavirus 2 (SARS-CoV-2) virus and demonstrate the assay on clinical samples using a simple and accessible point-of-care (POC) instrument. We characterized the assay by dipping swabs into synthetic nasal fluid spiked with the virus, moving the swab to viral transport medium (VTM), and sampling a volume of the VTM to perform the RT-LAMP assay without an RNA extraction kit. The assay has a limit of detection (LOD) of 50 RNA copies per μL in the VTM solution within 30 min. We further demonstrate our assay by detecting SARS-CoV-2 viruses from 20 clinical samples. Finally, we demonstrate a portable and real-time POC device to detect SARS-CoV-2 from VTM samples using an additively manufactured three-dimensional cartridge and a smartphone-based reader. The POC system was tested using 10 clinical samples, and was able to detect SARS-CoV-2 from these clinical samples by distinguishing positive samples from negative samples after 30 min. The POC tests are in complete agreement with RT-PCR controls. This work demonstrates an alternative pathway for SARS-CoV-2 diagnostics that does not require conventional laboratory infrastructure, in settings where diagnosis is required at the point of sample collection.

## Significance

An important limitation of current assays for the detection of SARS-CoV-2 stems from their reliance on time-consuming, labor-intensive, and laboratory-based protocols for viral isolation, lysis, and removal of inhibiting materials. While RT-PCR remains the gold standard for performing clinical diagnostics to amplify the RNA sequences, there is an urgent need for alternative testing platforms that are rapid, accurate, simple, and portable. Here, we demonstrate isothermal RT-LAMP nucleic acid-based detection of SARS-CoV-2 with an additively manufactured cartridge and a smartphone-based instrument for testing that can be performed at the point of sample collection.

Since severe acute respiratory syndrome coronavirus 2 (SARS-CoV-2) jumped from an animal reservoir to humans in December 2019, the acute respiratory disease named COVID-19 has rapidly spread across the world, bringing death, illness, disruption to daily life, and economic losses to businesses and individuals (1–3). The rapid development of the COVID-19 pandemic highlights shortcomings in the existing laboratory-based testing paradigm for viral diagnostics (4). The fundamental limitations of current diagnostic assays for viral pathogens stem from their reliance upon PCR analysis, which requires temperature cycling as well as labor-intensive, laboratory-based protocols for viral isolation, lysis, and removal of inhibiting materials. Although, recently, the RT-qPCR technique has demonstrated detection of SARS-CoV-2, bypassing the need for RNA isolation/purification starting from a saliva sample (5), the PCR technique is not easily adaptable for point-of-use detection, due to the need for temperature cycling. While PCR remains the proven gold standard for clinical diagnostics, there is an urgent need for other approaches that are sufficiently low cost and rapid to provide diagnosis at the point of use.

In addition to the Centers for Disease Control and Prevention (CDC) RT-PCR SARS-CoV-2 test (6), other diagnostic tests have become available, including the Cepheid Xpert Xpress SARS-CoV-2 test (7), the Abbott ID NOW COVID-19 test (8), the Color SARS-CoV-2 loop-mediated isothermal amplification (LAMP) Diagnostic Assay (9), and others (10–20). The Cepheid SARS-CoV-2 test can provide positive and negative results for the detection of SARS-CoV-2 in ∼30 and 45 min, respectively (21). However, this test requires the GeneXpert system, of which there are only 5,000 systems available in the United States (22). The test also requires RNA extraction as a separate step from amplification and detection, which is a constraint on scalability and may limit availability as demand increases for critical supplies (23). The Abbott ID NOW isothermal amplification technology claims the delivery of positive results in less than 15 min while offering a device with portable size and weight. However, this test also requires a specialized instrument with well-documented availability issues (24–26). There may also be issues with accuracy of this test, although claims about accuracy remain under review (27). Another isothermal technology with good performance is the SARS-CoV-2 LAMP Diagnostic Assay from Color Genomics, with a limit of detection (LOD) of around 0.75 copies of viral RNA per microliter 100% positive and negative agreement for 543 patient samples (9). The assay is also interesting for its colorimetric readout that could enable novel applications and form factors. However, the sample preparation includes a bead-based RNA extraction step that can increase the cost and time required to perform the assay.

Diagnostics based on LAMP amplification are a compelling alternative to PCR, because LAMP can be performed without the need for commercial thermocyclers, resulting in decreased time to result (28). Very importantly, the simplicity of isothermal amplification and the absence of separate steps for viral extraction allow for translation to a simple point-of-use device based on disposable cartridges (29). In addition, RT-LAMP has advantages over RT-PCR for targeting sequences, due to its robustness against inhibitors (30, 31) as well as its high specificity, using four to six primers that identify six to eight regions on the template for amplification (28).

This paper demonstrates RT-LAMP detection of SARS-CoV-2 POC using a simple and portable diagnostic system based on an additively manufactured three-dimensional (3D) cartridge and a smartphone-based optical reader. We demonstrate the point-of-care (POC) diagnostic system by detecting SARS-CoV-2 in 10 viral transport medium (VTM) clinical samples without using any other external equipment for the sample/reagent mixing, amplification, or readout. We also present detailed characterization of the assay development using VTM, synthetic spiked nasal solutions, and the analysis of clinical VTM samples from patients.

## Results

### POC System Approach.

**Fig. 1.**
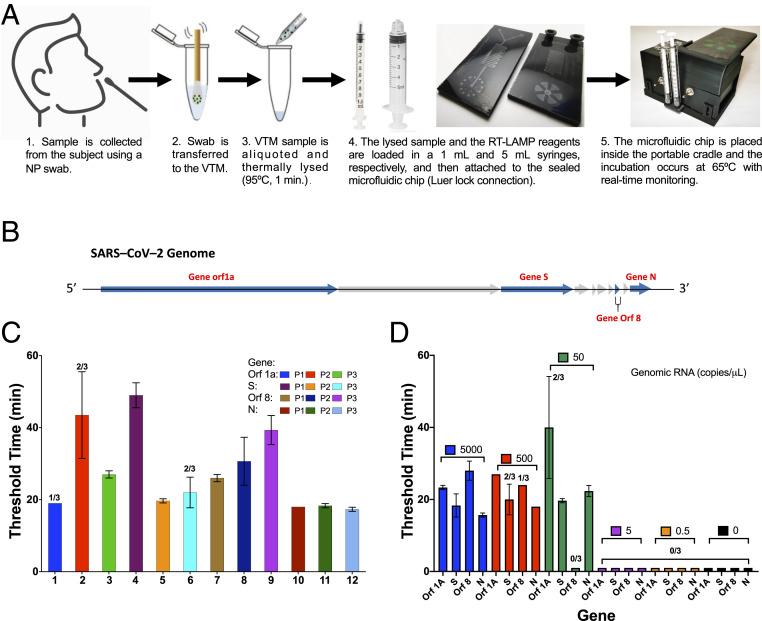
Validation of three LAMP primer sets for four different SARS-CoV-2 gene targets. (*A*) Workflow for the detection of SARS-CoV-2 using our portable POC device. (*B*) SARS-CoV-2 genome outline and four gene targets for primer design. (*C*) Comparison of positive amplification threshold time for four genes (500 copies per μL, *n* = 3) using primer set 3 for gene Orf 1a, primer set 2 for gene S, primer set 2 for gene Orf 8, and primer set 1 for gene N. (*D*) Amplification threshold times (*n* = 3) for detection of different concentration of genomic RNA using primer set 3 for gene Orf 1a, primer set 2 for gene S, primer set 2 for gene Orf 8, and primer set 1 for gene N. The best detection limit was 50 copies per μL attained using gene N primer set 1. The bar graphs show mean and SD.

The complete workflow from sample collection to the analysis using our portable device is shown in Fig. 1*A*. First, the sample is acquired from the patient using a nasopharyngeal (NP) swab. Then, the swab is transported to VTM solution and gently agitated to transfer the viruses from the swab into the VTM. Third, the swab is discarded, and aliquots from the VTM are thermally lysed (95 °C, 1 min). Next, the lysed sample and the RT-LAMP reagents are loaded in 1- and 5 mL-syringes, respectively, then the syringes are attached to the microfluidic cartridge, and the lysed sample and RT-LAMP reagents are simultaneously injected into the cartridge. Finally, the cartridge is placed inside the portable smartphone cradle, and the nucleic acid amplification with intercalating fluorescent dye occurs at 65 °C. Real-time monitoring of the fluorescence emission generated during amplification is performed using a smartphone camera, and image analysis provides the time at which amplification occurred.

### Primer Design and Assay Characterization with SARS-CoV-2 Genomic RNA.

To develop a sensitive and specific RT-LAMP assay for the detection of SARS-CoV-2, we designed sequence-specific primers for four genes from the SARS-CoV-2 viral genome. Using basic local alignment search tool for nucleotides (BLASTn) analysis, we identified genes Orf 1a, S, Orf 8, and N for primer design, which code for the Orf1ab polyprotein, surface glycoprotein, Orf 8 protein, and nucleocapsid phosphoprotein, respectively (Fig. 1*B*). Target regions Orf 1ab, S, and Orf 8 were selected because they showed the least similarity with other coronavirus sequences such as SARS-CoV-1 and Middle East respiratory syndrome-CoV (32). The gene N target region was selected due to its overlap with the region used for primer design in currently CDC- and Food and Drug Administration-approved assays for COVID-19 (33). Three primer sets for each of the four selected genes were generated using Primerexplorerv4 (https://primerexplorer.jp/e/), and RT-LAMP experiments using SARS-CoV-2 genomic RNA were performed in a standard thermocycler at a fixed temperature. Fig. 1*C* shows the threshold times for detection of SARS-CoV-2 genomic RNA (500 copies per μL) using primer sets for gene Orf 1a, gene S, gene Orf 8, and gene N. The amplification curves (raw data) can be found in *SI Appendix*, Fig. S1. The best primer set for each gene (primer set 3 for gene Orf 1a, primer set 2 for gene S, primer set 2 for gene Orf 8, and primer set 1 for gene N) was selected based on lowest threshold time and used for LOD analysis. All of the primer sequences tested are shown in *SI Appendix*, Table S1.

Next, we compared the LOD of the four selected primers sets by amplifying serial dilutions of SARS-CoV-2 RNA (Fig. 1*D*). The LOD for RNA using the gene Orf 1a primer set was 500 copies per μL with only 2/3 replicates giving amplification for 50 copies per μL of SARS-CoV-2 RNA. The detection limit for gene S and gene Orf 8 primers was of 5,000 copies per μL of RNA, as not all replicates amplified for 500 copies per μL of sample. The reactions performed with gene N primers demonstrated the lowest LOD and fastest amplification times, with 50 copies per μL amplifying within 25 min of the reaction start. The amplification curves (raw data) can be found in *SI Appendix*, Fig. S2. Based on these results, we chose primer set 1 targeting gene N as the final primer set for our RT-LAMP assay for SARS-CoV-2 detection.

### Assay Characterization Using Inactivated SARS-CoV-2 Viruses and Simulated Clinical Workflow.

**Fig. 2.**
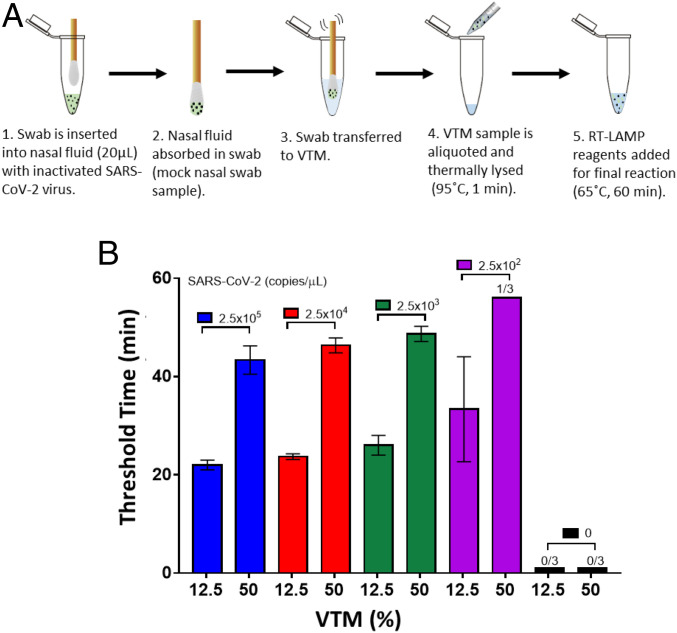
Detection of SARS-CoV-2 virus from mock NP swab samples transported to VTM. (*A*) Process flow for viral detection from a mock NP swab. A swab is inserted into a tube with virus-spiked nasal fluid and absorbs the fluid. After vigorously mixing the swab in 100 μL or 500 μL of VTM, an aliquot of the VTM sample is thermally lysed at 95 °C for 1 min. The RT-LAMP reagents are added to the lysed viral sample, and the reaction is conducted at 65 °C for 60 min. (*B*) Amplification threshold times (*n* = 3) for viral detection in a 16-μL reaction with 12.5% and 50% VTM sample per reaction from a 500-μL VTM sample.

For diagnostic testing of SARS-CoV-2, the current clinical workflow includes collecting NP/nasal specimens using swabs, which are immediately transferred into a sterile transport tube, containing 2 mL to 3 mL of VTM, for storage until diagnostic assays can be performed (34). To evaluate the performance of our detection assay in the current clinical workflow, a protocol with simulated NP/nasal swab samples was developed as shown in Fig. 2*A*.

Before testing this protocol, the effects of the thermal lysis for detecting inactivated SARS-CoV-2 in nasal fluid and in VTM were characterized. These results are summarized in *SI Appendix*, Figs. S3–S6. Next, to evaluate our assay in the current clinical workflow, commercial swabs (Puritan sterile polyester tipped applicators, 25-800D 50) were introduced into purchased nasal solution spiked with known virus concentrations. The swab was transferred to VTM and gently agitated in the solution to transfer the viruses from the swab into the VTM. The swab was thereafter discarded, and aliquots from the VTM were taken to perform thermal lysis at 95 °C for 1 min. Finally, RT-LAMP reagents were added, and the final reaction was performed at 65 °C for 60 min. We transferred the mock swabs to 100 and 500 μL of VTM to evaluate VTM volume effects on swab viral load transfer efficiency. For each condition above, we performed LOD tests with 12.5% and 50% VTM per reaction. The results obtained using 500 μL of VTM can be seen in Fig. 2*B* (raw amplification curves can be found in *SI Appendix*, Fig. S7). The results obtained using 100 μL of VTM can be found in *SI Appendix*, Fig. S8.

For 100 μL of VTM, the LOD remained 2.5E4 copies per μL of virus in the starting nasal fluid for both 12.5% and 50% VTM in reaction, even though 50% VTM reactions showed delayed amplifications. The above detection limit in nasal fluid amounts to 5E3 copies per μL of virus in VTM after swab transfer. This is two orders of magnitude greater than the 50 copies per μL detection limit of viruses directly spiked in VTM and indicates inefficient viral transfer from swab to 100 μL of VTM solution, which is present in all swab-based sample collection processes. This inefficiency likely arises due to inadequate adsorption of viruses into the swab, and subsequent inadequate release of the viruses into the VTM. The low interfacial contact area between the swab and the VTM due to VTM volume could also play a role in the poor release of the viruses into the VTM.

For 500 μL of VTM with 12.5% and 50% VTM in reaction, the LOD improved to 250 copies per μL and 2.5E3 copies per μL of virus in nasal fluid, respectively. The above detection limits in nasal fluid amount to 10 copies per μL and 100 copies per μL of virus in VTM after swab transfer, which are comparable to the control experiments where viruses were directly spiked in VTM. This indicates efficient viral release in 500 μL of VTM, with transfer efficiency close to 100%. Three swab replicates were performed for each condition.

### Assay Development for SARS-CoV-2 Detection from Patient Samples.

**Fig. 3.**
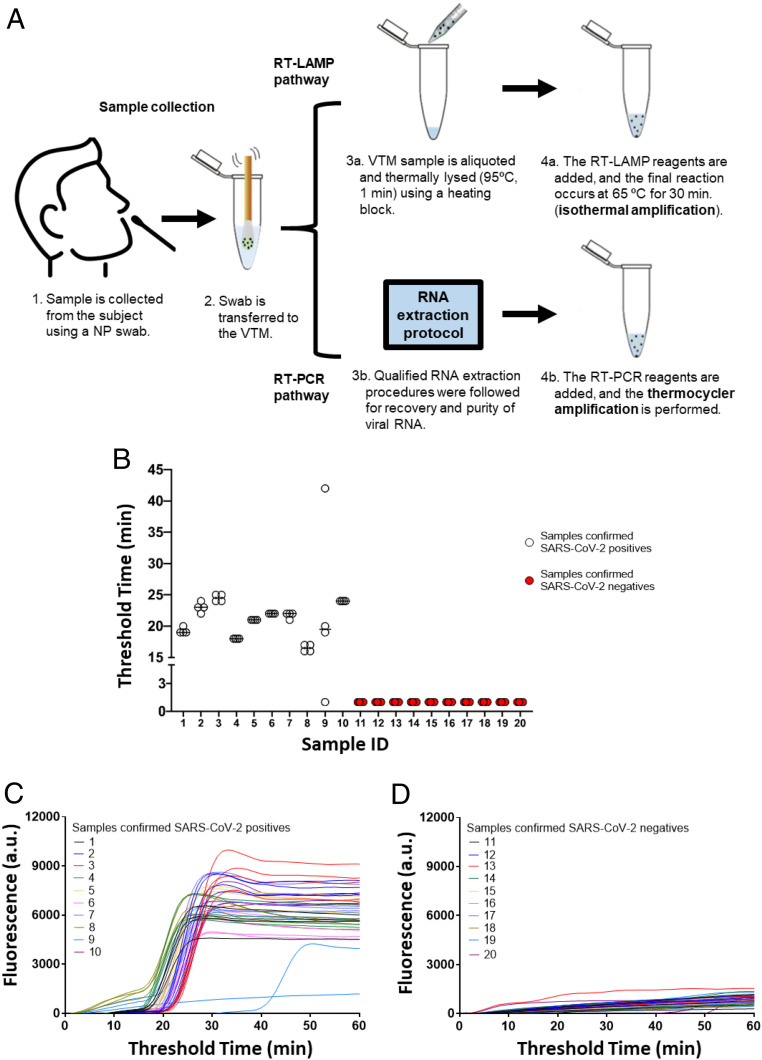
Detection of SARS-CoV-2 virus from VTM clinical samples. (*A*) Process flow for viral detection from VTM clinical samples. The sample is collected from the patient using an NP swab. After the sample is transferred to the VTM (step 2), an RT-PCR test is performed, and the results are used as control. The discarded VTM is frozen for transfer and storage. After thaw, aliquots are thermally lysed (step 3b) before the RT-LAMP is conducted (65 °C, 60 min). RT-LAMP pathway does not require of RNA extraction. (*B*–*D*) Assessment of clinical samples (*n* = 4). VTM samples from 10 SARS-CoV-2−positive and 10 SARS-CoV-2−negative patients (as judged by RT-PCR control test with RNA extraction) were analyzed using the developed RT-LAMP assay. (*B*) Undetermined Ct values are plotted at 1. (*C* and *D*) Raw fluorescence data (*n* = 4) for SARS-CoV-2 detection from VTM clinical samples.

We also characterized our RT-LAMP reaction using 20 clinical samples (10 known positives and 10 known negatives) obtained from OSF Healthcare, Peoria, IL, through an approved instituional review board (OSF Peoria IRB # 1602513 through the University of Illinois College of Medicine with waiver for consent). The samples received were VTM discards prior to the RNA purification step. Along with the samples, we also received the results of the RT-PCR tests performed by OSF Healthcare. Fig. 3*A* shows a process flow schematic of viral detection from VTM clinical samples (RT-LAMP assay and RT-PCR control). Samples were collected following clinical gold standard techniques (using an NP swab) and were frozen.

All 10 samples identified as positive by RT-PCR and all 10 samples identified as negative by RT-PCR were also positive and negative by our RT-LAMP assay (Fig. 3*B*). Thus, the sensitivity and specificity of our assay for SARS-CoV-2 was 100%, with false negative and false positive rates of 0%. The amplification curves (raw data) can be found in Fig. 3 *C* and *D*. A table, showing the threshold times (RT-LAMP assay) and the Ct values (control) are shown in *SI Appendix*, Table S2.

### SARS-CoV-2 Detection from Patient Samples in Portable Reader and Additively Manufactured Cartridges.

**Fig. 4.**
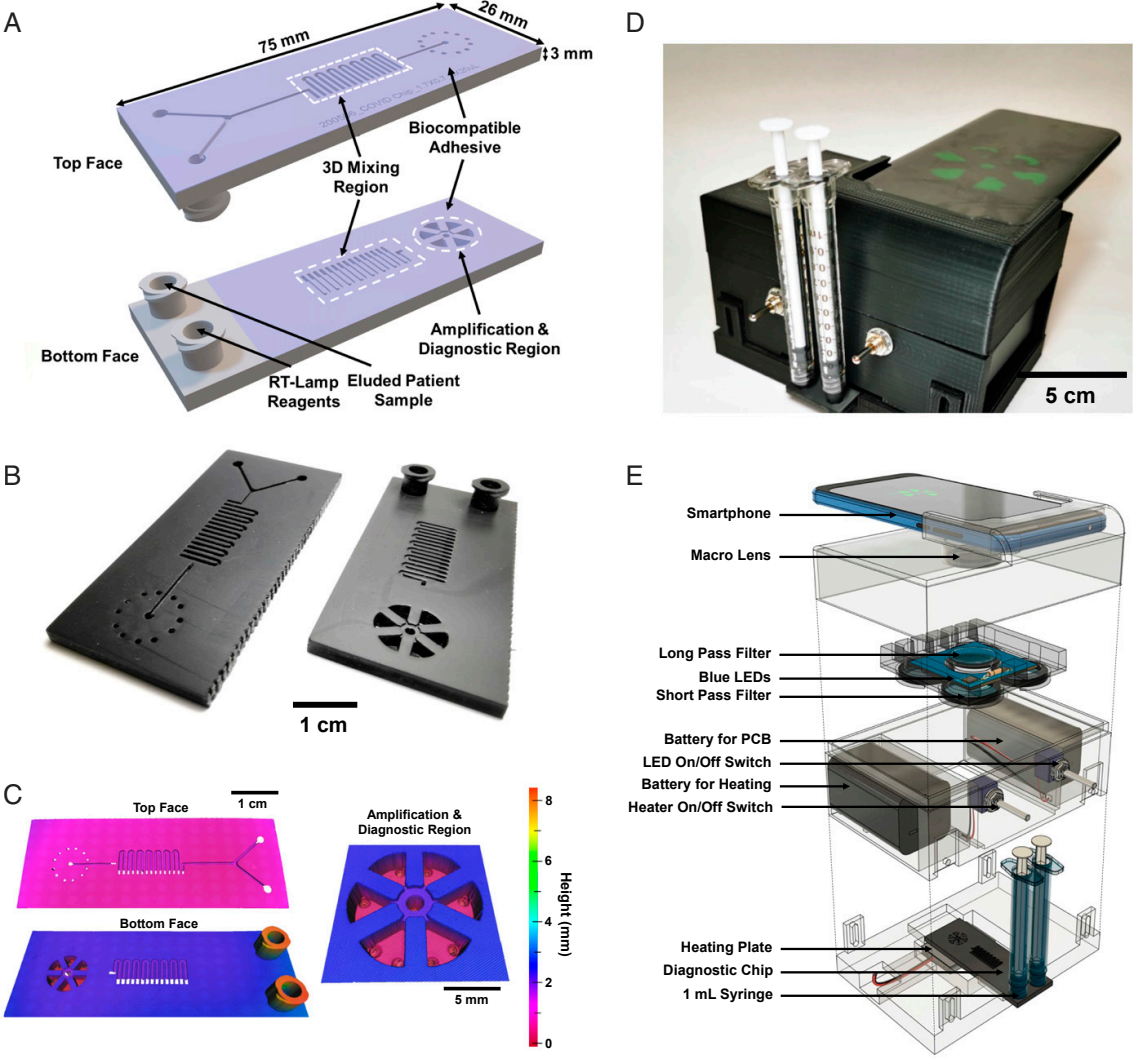
Additively manufactured microfluidic cartridge and handheld POC instrument. (*A*) Diagram of the microfluidic diagnostic cartridge used for rapid detection of SARS-CoV-2 in VTM. The fluid inlet ports mate with syringes that inject either RT-LAMP reagents or thermally lysed patient sample into a 3D serpentine mixing region before filling the amplification and diagnostic region. (*B*) Photographs of the disposable microfluidic cartridge. (*C*) The 3D scans of the microfluidic cartridge and magnified view of the detection pools in the amplification and diagnostic region of the cartridge. (*D*) Photograph of the instrument used for rapid detection. (*E*) Schematic of the handheld POC instrument showing components in an exploded view. A smartphone images the cartridge, while isothermal heating and illumination are battery powered. Optical components integrated with the instrument match the excitation and emission characteristics of the fluorescent signal.

Finally, we demonstrate detection of SARS-CoV-2 viruses from clinical samples using the VTM samples from patients (OSF Healthcare) and using our portable handheld reader. In these tests, the samples were loaded manually without the use of pumps. The portable handheld reader included heating elements and optics necessary for performing and recording the reaction. Fig. 4 *A*–*C* shows a schematic diagram, photo, and mechanical drawings of the diagnostic microfluidic cartridge (top and bottom plane of the serpentine mixing channels) used for rapid detection of SARS-CoV- 2 in VTM. The amplification and diagnostic regions include six pie-shaped amplification chambers. The cartridge had 3D serpentine microfluidic channel features for mixing of the viruses in the VTM sample with the amplification reagents. After the mixing, the final reaction mix enters the amplification reservoirs. The reservoirs were specifically designed to allow uniform filling of all of the chambers, as can be seen in Movie S1. After the amplification chambers were completely loaded, they were sealed with biocompatible adhesive, and the cartridge was inserted into the reader for the final reaction. The integrated heater was set at 65 °C, and a smartphone was used to perform the imaging. Fig. 4*D* shows an optical image of the reader with inserted cartridge. RT-LAMP reagents and virus-spiked VTM sample were loaded using syringes through Luer lock-compatible inlet ports. Fig. 4*E* shows the schematic of the handheld POC instrument showing components in an exploded view, detailing the components of the reader. While a smartphone images the cartridge, the isothermal heating and illumination are battery powered. LEDs and emission filters were selected to match the excitation and emission wavelengths of the intercalating fluorescent dye.

Before testing the clinical samples, an initial assessment of the platform was performed by spiking inactivated SARS-CoV-2 (5,000 copies per μL) and negative control (0 copies per μL) in VTM. The baseline-subtracted real-time fluorescence images of amplification on the cartridge for 5,000 copies per μL of virus in VTM and negative control (VTM only) are shown in *SI Appendix*, Fig. S9. Likewise, Movie S1 also shows time-stamped video of amplification on the cartridge for 5,000 copies per μL of virus in VTM and negative control (VTM only). Using this spiked sample, a positive detection was absorbed in as little as 30 min from the start of the reaction.

From the VTM clinical samples tested off-cartridge, five positive and five negative samples were selected for the characterization of our device. The selection criteria for these five positive samples was based on the C_t_ values reported from the clinical RT-PCR−based controls. Although some VTM clinical samples had C_t_ values in the range from 11 < C_t_ < 20, we selected five positive samples with C_t_ values in the range of 20 to 30, as some studies have reported clinical sample Ct values in this range. For instance, the C_t_ values of 17 symptomatic patients in relation to the day of onset of any symptoms were reported (35). This work reported C_t_ values in the range of 20 to 30 during the first 12 d after the onset of symptoms (nasal swabs). Likewise, this study included the analysis of one patient with no reported clinical symptoms. In this case, the asymptomatic patient was tested positive on days 7, 10, and 11 after contact with another confirmed SARS-CoV-2−infected patient (C_t_ values: 22 to 28, nasal swab). In another example, 16 critically ill SARS-CoV-2 patients were tested, and the median and third quartile C_t_ values were in the range of 20 to 30 (from nasal swab) (36).

**Fig. 5.**
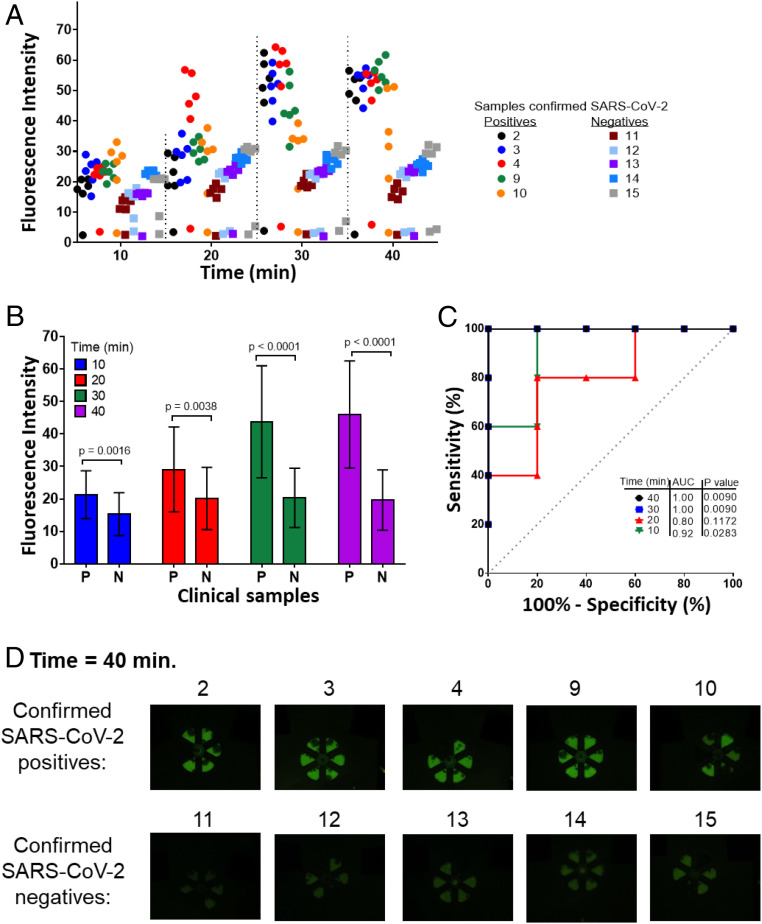
Rapid detection of SARS-CoV-2 in VTM clinical samples using an additively manufactured microfluidic cartridge and handheld POC instrument. (*A*) Fluorescence intensities of real-time RT-LAMP on the additively manufactured amplification chip at different time points. The developed device can clearly differentiate all of the positives samples from the negatives as fast as in 30 min (*n* = 6). (*B*) A *t* test shows that the fluorescence intensity of the positive samples (P) is statistically significant in comparison with the negative samples (*N*). (*C*) ROC curves analyzed for the four time conditions. For each time point, the positive samples were analyzed against the negative samples. (*D*) Fluorescence images of real-time RT-LAMP SARS-CoV-2 analysis at the endpoint of detection on the additively manufactured amplification chip.

Fig. 5 shows the assessment of 10 clinical samples using our RT-LAMP−based POC system, which show complete agreement with the RT-PCR measurements. Our platform provided real-time fluorescence amplification images, without the requirement of any other external equipment. For the analysis of each clinical sample, one microfluidic cartridge was used. Likewise, each test included the analysis of six subsamples (six pie-shaped amplification chambers). The fluorescence images were analyzed using Image J software, and the fluorescence intensity of each replicate was plotted as a function of time (Fig. 5*A*). The *t* tests were performed to demonstrate that the fluorescence intensity of the positive samples is statistically significant in comparison with the negative samples (Fig. 5*B*). Receiver operating characteristic (ROC) curves were plotted to compare positives samples against negative samples for each time point (Fig. 5*C*). AUC for 40 and 30 min was 1.00 in both cases, showing that our system can correctly differentiate positive from negative samples after 30 min. Fig. 5*D* shows the amplification images obtained for all of the samples tested at the endpoint of detection (40 min). Real-time amplification images can be seen in *SI Appendix*, Fig. S10.

## Discussion

The current gold standard method for the detection of SARS-CoV-2 virus from NP swabs is based on RT-PCR, which also requires laboratory-based protocols for viral extraction and concentration. Most commercially available COVID-19 diagnostic tests in the United States, Europe, and Asia are benchtop-type systems intended for laboratory use and are not tailored for portability and point-of-use applications (37). While the current laboratory-based paradigm for SARS-CoV-2 is scalable and can be automated for high throughput, there is an urgent need for alternatives that expand the options for testing at POC and in low-resource settings. While testing resources are available in some densely populated and wealthy regions, some regions do not have easy access to laboratory-based testing that requires specialized equipment, infrastructure, and expertise. To broaden this access, there is a need for technologies that are fast, portable, and low cost, while also capable of delivering detection limits that are comparable to laboratory methods.

In this paper, we demonstrate rapid (<40 min) detection of virus directly from viral transport media NP swabs using a portable hand-held reader and additively manufactured cartridges. This POC device uses an isothermal RT-LAMP assay for rapid and cost-effective detection of SARS-CoV-2. Using clinical samples, the presence of the virus was interrogated in 10 clinical samples (five confirmed positives and five confirmed negatives). Our system can distinguish positive from negative samples after 30 min of the on-cartridge reaction, demonstrating complete agreement with RT-PCR measurements of the same sample. A key step that simplifies the assay is thermal lysis, which efficiently disrupts the viruses to release the RNA for amplification, while also inactivating the nucleases that are present in unpurified samples (38). Thermal lysis has advantages over solution-based sample treatments, which dilute the sample and can result in reduced sensitivity. This fully portable approach can detect the virus rapidly while eliminating the need for an RNA extraction kit and related instrumentation. This approach could enable the scalable deployment of COVID-19 diagnostics without laboratory-grade infrastructure and resources, especially in settings where diagnosis is required at the point of collection, such as schools, facilities that care for the elderly or disabled, or sporting events.

The POC instrument is designed for low cost, accessibility, and the potential for scale-up. Because the entire assay can be conducted within the cartridge, the principle of operation is very simple and can be performed with minimal training. The instrument was constructed from commercially available components and a housing that was easily made on a consumer 3D printer. The optical detection can be performed using nearly any modern smartphone. The disposable cartridge, which normally presents significant technical challenges for manufacturing scale-up, was made using high-speed production-grade additive manufacturing equipment and requires no additional work to be made at scale using these methods.

Although there have been recent reports of RT-LAMP−based assays to detect SARS-CoV-2 (39, 40), to the best of our knowledge, no previous study has shown detection of SARS-CoV-2 using a handheld portable instrument with an integrated disposable cartridge without RNA extraction. Other relevant technologies, such as the assay from Color Genomics, report high throughput with a low LOD (0.75 copies per μL) but require a bead-based RNA extraction step (9). Likewise, a recent paper reported the development of an RT-LAMP/Cas12 assay for detection of SARS-CoV-2 with an LOD = 10 copies per μL, but also requires RNA extraction (41).

On the benchtop (outside the cartridge), our assay tested on 20 patient samples had 100% accuracy. As demonstrated in Fig. 3 *B*–*D*, the sensitivity [defined as True Positives/(True Positives + False Negative) and specificity (defined as True Negatives/(True Negatives + False Positives)] of our benchtop assay was 100% in the samples tested. All 10 samples identified as positive by RT-PCR and all 10 samples identified as negative by RT-PCR were also positive and negative, respectively, via our assay. The 20 samples were analyzed with four 2-μL replicates each, showing high reproducibility. The RT-LAMP assay bypasses the need for RNA isolation/purification, reducing the overall cost and time of the assay. However, bypassing the RNA extraction and concentration step, along with the small sample volume (2 μL) used in each reaction, could explain the results obtained in sample 9, where one replicate did not amplify, and another replicate amplified later in time. In the future, use of a larger assay volume could obviate sampling issues and improve the sensitivity.

The POC assay demonstrated a 50 copies per μL LOD with genomic RNA and inactive whole viruses in buffer, and no loss of sensitivity in the VTM. Published reports studying the viral load of SARS-CoV-2 in clinical samples suggest that this LOD corresponds to clinical needs. For example, a study performed on 3,303 patients who tested positive for SARS-CoV-2 estimated the viral load to be in the range of 1 copy per μL to 10^8^ copies per μL, with the majority of the samples in the range of 10^4^ to 10^8^ copies per μL (42). In another study with 1,145 hospitalized SARS-CoV-2 positive patients (average age of 64.6 y), the median viral load was 1,440 viral copies per μL (43). These viral loads can easily be measured using our POC device.

Finally, there are now very recent reports of the use of saliva as an alternative to the NP swab collection process. These reports have demonstrated the ability to directly detect SARS-CoV-2 from patient saliva samples using RT-PCR (5) and RT-LAMP (40) without RNA purification steps, using a larger sample volume (>60 μL). The promising results of the present study could likely be extended for use with saliva samples for noninvasive, portable, rapid, and scalable testing for COVID-19.

## Materials and Methods

### SARS-CoV-2 Genomic RNA and Viruses.

Genomic RNA for SARS-Related Coronavirus 2 (Isolate USA-WA1/2020), NR-52285, was obtained from BEI Resources. These genomic RNA vials were stored at −80 °C, and stock volumes were either used directly for experimentation or diluted to the correct concentration in TE Buffer. For experiments using virus, Heat Inactivated SARS-Related Coronavirus 2, NR-52286, was obtained through BEI Resources. These stocks were aliquoted and stored at −80 °C. Stock volumes were either used for direct experimentation or diluted in TE Buffer or Viral Transport Media to the correct concentrations.

### Primer Sequences and Primer Validation Reactions.

Primer sequences for the RT-LAMP reactions were synthesized by Integrated DNA Technologies and are listed in *SI Appendix*, Table S1. Primerexplorerv4 (https://primerexplorer.jp/e/) was used to design all sets of RT-LAMP primers for SARS-CoV-2 RNA and virus. The sequence for SARS-CoV-2 virus was obtained from the National Center for Biotechnology Information database (GenBank number MN988713.1).

In total, 12 sets of primers (three sets of primers each for four different gene targets) were tested with SARS-CoV-2 genomic RNA as template to determine the best primer set for each gene that detected 500 copies of RNA per μL with the lowest threshold time. Primer set 3 for gene Orf1a, primer set 2 for gene S, primer set 2 for gene Orf 8, and primer set 1 for gene N were selected. Thereafter, RT-LAMP assays with the four selected primer sets were conducted on 10-fold serially diluted RNA to determine the detection range. The gene N targeting primer set (primer set 1) showed detection of 50 genomic copies per μL of SARS-CoV-2 genomic RNA as the limit and was therefore selected as the working primer set used in the downstream RT-LAMP assays.

### Genomic RNA and Virus in Buffer Detection in RT-LAMP Reactions.

The following components comprised the RT-LAMP assay: 4 mM of MgSO_4_ (New England Biolabs), 1× final concentration of the isothermal amplification buffer (New England Biolabs), 1.025 mM each of deoxyribonucleoside triphosphates, and 0.29 M Betaine (Sigma-Aldrich). Individual stock components were stored according to the manufacturer’s instructions, and a final mix including all of the components was freshly created prior to each reaction. Along with the buffer components, a primer mix consisting of 0.15 μM F3 and B3, 1.17 μM forward Inner primer (FIP) and backward inner primer (BIP), and 0.59 μM of LoopF and LoopB was added to the reaction. Finally, 0.47 U/μL BST 2.0 WarmStart DNA Polymerase (New England Bioloabs), 0.3 U/μL WarmStart Reverse Transcriptase (New England Biolabs), 1 mg/mL bovine serum albumin (New England Biolabs), and 0.735× EvaGreen (Biotium) were included in the reaction. EvaGreen dye is a double-stranded DNA intercalating dye. After addition of the template, the final volume of the reaction was 16 μL. All reactions with genomic RNA template in TE Buffer included 2 μL of template to make the final reaction volume of 16 μL.

All of the off-chip RT-LAMP assays were carried out in 0.2-mL PCR tubes in an Eppendorf Mastercycler realplex Real-Time PCR System at 65 °C for 60 min. Fluorescence data were recorded every 1 min after each cycle of the reaction. Three repeats were done for each reaction.

Reactions done with heat-inactivated viruses included a thermal lysis step. First serially diluted in TE Buffer, viral samples were then thermally lysed in a heater at 95 °C for 1 min prior to their addition into the final reaction mix.

All of the RT-LAMP reactions consisted of nontemplate negative controls that were included in all of the datasets.

### Detection of Virus Spiked in Nasal Fluid.

Nasal fluid was commercially obtained from Innovative Research, and it was confirmed that the fluid samples were obtained prior to the COVID-19 pandemic. Serially diluted SARS-CoV-2 heat-inactivated viruses in TE Buffer were spiked directly into nasal fluid such that the viral sample concentration in nasal fluid ranged from 50 PFU/μL to 0.005 PFU/μL (5E5 to 50 copies per μL). The virus in nasal fluid sample was then thermally lysed at 95 °C for 1 min prior to adding the RT-LAMP reagents mix for a total reaction volume of 16 μL. The sample volumes were varied such that the spiked nasal fluid sample volume was 12.5%, 25%, or 50% of the total reaction volume. One reaction was conducted in the same format, in which total reaction volume was 96 μL, of which 48 μL was the sample volume. In all of these reactions, the concentrations of all other reactions’ components were maintained as mentioned above for the 16-μL reaction.

### Detection of Virus in Viral Transport Media.

Moreover, reactions were done with heat-inactivated viruses in VTM. CDC-compliant VTM was obtained from Redoxica (VTM-500ML), aliquoted, and stored in 4 °C away from direct light. Viruses were serially diluted in VTM to starting sample concentrations ranging between 0.5 PFU/μL and 0.005 PFU/μL (5,000 copies per μL to 50 copies per μL). Then, the samples were thermally lysed at 95 °C for 1 min prior to adding the RT-LAMP reagents mix for a total reaction volume of 16 μL. Two different sample volumes were tested in which the virus in VTM sample was either 12.5% (2-μL sample) or 50% (8-μL sample) of the total reaction volume. In these reactions, the concentrations of the buffer, primer, polymerase, and other reaction components were kept constant as mentioned above within the 16-μL reaction.

### Detection of Virus in Nasal Fluid Collected on NP Swabs.

CDC-approved NP swabs (Sterile Polyester Tipped Applicators) were commercially obtained from Fisher Scientific. As described above, serially diluted SARS-CoV-2 heat-inactivated viruses in VTM were spiked directly into nasal fluid such that the viral sample concentration in nasal fluid ranged from 25 PFU/μL to 0.0025 PFU/μL (2.5E5 to 250 copies per μL). Spiked nasal fluid (20 μL) was first absorbed by an NP swab and then transferred into 100 μL of VTM. The swab was mixed in the VTM for 30 s to 1 min for viral transfer from the NP swab. After removing the swab, the VTM sample was distributed into sample aliquots and thermally lysed at 95 °C for 1 min. Finally, the rest of the reagents for the RT-LAMP reaction were added to the sample for a total reaction volume of 16 μL.

### Off-Chip Amplification Data Analysis.

The off-chip RT-LAMP fluorescence curves and amplification threshold bar graphs were analyzed using a MATLAB script and plotted using GraphPad Prism 8. For each curve, the threshold time was taken as the time required for each curve to reach 20% of the total intensity. The amplification threshold bar graphs show the mean and SD of three samples.

### Additively Manufactured Microfluidic Cartridge.

Fig. 4*C* shows the disposable polymer cartridge developed for the rapid detection of SARS-CoV-2 in VTM. The 3D design consists of microfluidic channels on both front and back sides, connected by 1.7 mm × 0.7 mm^2^ through-holes at the end of each serpentine microchannel. The chip was designed and additively manufactured as a single component to complete three functions on-chip. First, thermally lysed patient sample and RT-LAMP reagents are injected through the female Luer lock connectors from two separate syringes without the use of microfluidic pumps. Each access port is directly connected to the continuous 3D flow pathway by a Y-shaped inlet region. Then the sample flows through the 3D micromixer region, where the flow takes a vertical turn from one face to the other face between each horizontal U-turn. There are seven serpentine channels on the top face and eight on the bottom face, with each serpentine microchannel being 0.7 mm wide, 0.4 mm deep, and 8 mm long. The alternating horizontal and vertical U-turns enhance mixing and allow for dense packing of the mixing structure. Finally, the fluid flows into six reservoirs that radially surround the flow channel furcation. These detection reservoirs located at the end of the chip are designed to contain a volume of ∼20 μL per chamber. The amplification chambers have a 0.5-mm-thick wall and two 1.1-mm-diameter outlet holes to remove excess air during filling.

The cartridge was fabricated from rigid polyurethane (RPU) on a Carbon M2 printer using standard process settings, washed, and then cured. The print orientation for this part was the bottom face of the chip attached to build tray, and Luer lock fluid ports facing upward. After fabrication, the cartridge was washed again in water and dried using pressurized air. The front and back sides of the cartridge were covered with transparent biocompatible tape (ARSeal 90880, Adhesive Research) to seal the chip. The transparent tape allows for visual inspection during filling and optical imaging during detection. Following tape application, two holes were made using a needle in the tape for each reservoir; these holes serve as air outlets during filling.

### Cradle Fabrication and Smartphone-Based Fluorescence Imaging.

The microfluidic diagnostic cartridge mates with an instrument shown in Fig. 4 *D* and *E*. We used a smartphone (Huawei P30 Pro, Huawei) to detect the fluorescence emission from on-chip LAMP assays. The instrument comprises four main parts to support optical, electrical, and heating components. The top part of the instrument holds the smartphone and aligns its camera with a macro lens (12.5×, Techo-Lens-01, Techo). The macro lens enables close-up imaging (∼50-mm imaging distance) of the chip. The second component, printed circuit board (PCB) and filter holder, is equipped with a long-pass filter (525 nm, 84-744, Edmund Optics), which allows only the emission light from the EvaGreen dye to reach the camera. A PCB is aligned with this long-pass filter and controls the illumination of the device. A total of eight LEDs (λpeak = 485 nm, XPEBBL, Cree) are mounted on the PCB in a circle to provide uniform illumination over the diagnostics area. Four short-pass filters (490 nm, 490SP RapidEdge, Omega Filters) covering each pair of LEDs are mounted on top of the PCB to excite the EvaGreen dye. The third component, namely, the main body of the cradle, carries two separate on−off switches and battery boxes to control the PCB and a heater. Finally, a self-regulating positive temperature coefficient (PTC) heater (12 V-80 °C, Uxcell) is located below the cartridge. A locking mechanism connects the main and bottom components, opens, and grants access to the chip to be inserted into the cradle. When inserted, only the diagnostics area of the chip is in contact with the heating plate that keeps the temperature at 65 °C during the amplification period.

The smartphone took photos of the chip at 10-min intervals in the first 30 min of assays, then at 2-min intervals to capture more amplification data points for another 10 min. The imaging settings of the smartphone were International Organization of Standardization (ISO) = 500 and exposure time = 0.5 s.

### Chip Loading of Viral Sample and RT-LAMP Assay Components.

For on-chip experiments with VTM clinical samples and spiked VTM samples, the sample was loaded into a 1-mL syringe. The RT-LAMP reagents were prepared off-chip and then loaded into a 5-mL syringe. All concentrations of reagents were maintained as in a 16-μL reaction. Both syringes were attached to the chip, and the sample was loaded on-chip without the use of a syringe pump. Once the sample and reaction reagents were loaded into the chip, the holes underneath the pie pools were sealed with a second double-sided adhesive layer to prevent leakage or evaporation during RT-LAMP incubation. The chip was placed into a portable cradle and clamped down with magnetic strips, such that the pie pools made good contact with the PTC heater throughout the RT-LAMP incubation step. The incubation occurred at 65 °C for 60 min with real-time monitoring.

### Chip Image and Data Analysis.

Fluorescence images were recorded with IP Webcam in smartphones and were saved in JPG format from which fluorescence intensity and baseline fluorescence was analyzed on Image J. The fluorescence intensity from all six pie pools was measured, averaged, and plotted on GraphPad Prism 8. ROC analysis was performed on GraphPad Prism 8.

### Supply Chain of Resources Required for Benchtop RT-LAMP Assay Compared to RT-PCR Test at Scale.

We quantified the resources required to scale up the RT-LAMP assay and compared it to the conventional RT-PCR test. For each test, we considered three scenarios in which the number of patient samples are 80, 800, or 8,000 (*SI Appendix*, *Supplementary Text*, Fig. S11, and Table S3 and Dataset S1).

## Supplementary Material

Supplementary File

Supplementary File

Supplementary File

## Data Availability

All study data are included in the article and *SI Appendix*.
