# Supplementary material for: Rapid isothermal amplification and portable detection system for SARS-CoV-2
Source: Proc Natl Acad Sci U S A. 2020 Aug 31;117(37):22727–35. doi: 10.1073/pnas.2014739117 (PMC7502724; doi:10.1073/pnas.2014739117)
Supplement: Supplementary File [file pnas.2014739117.sapp.pdf]

Supplementary Text

1. Thermal lysis

To evaluate the efficacy of our RT-LAMP assay to detect SARS-CoV-2, we prepared serial dilutions of spiked inactive SARS-CoV-2 viruses in our 16 μL RT-LAMP reactions and evaluate the effect of a short thermal lysis step on the detection limit of our assay (supplementary information **Figure S3**). When not including the thermal lysis step (the reaction was directly performed at 65°C), the detection limit was 500 copies/μL (0.005 PFU/μL) of starting inactivated virus concentration with only 2/3 replicates amplifying for 50 copies/μL starting concentration. The above reaction did not include any additional viral lysis step. When adding the short thermal lysis step (95°C, 1 minute) to the RT-LAMP protocol, we saw an improvement of 1 order in the LOD of the assay with a detection limit of 50 copies/μL. All three replicates amplified within 20 minutes of the reaction start (**Figure S3**). Henceforth, a 95°C, 1-minute thermal lysis was performed for all viral samples in the following experiments.

2. Using crude human nasal fluid samples

To characterize the robustness of our RT-LAMP assay, we spiked inactive SARS-CoV-2 viruses directly into purchased healthy human nasal fluid samples. Before the reaction, a thermal lysis step (95°C, 1 min) was performed.

To evaluate the effect of volume percentage of spiked nasal fluid per reaction on the detection limits and amplification times, we varied the spiked nasal sample per reaction from 12.5% to 50% of the total reaction volume (supplementary information, **Figure S4).** Surprisingly, we observed that the RT-LAMP reactions showed robust amplifications even for 50% nasal fluid per reaction. Moreover, we observed that higher sampling volumes of virus-spiked nasal fluid improved the detection limit of the assay from 5E5 copies/μL (for 12.5% nasal sample/reaction) to 5E3 copies/μL (for 50% nasal sample/reaction) in the final reaction. This is likely because the very viscous nasal fluid solution prevented effective pipette mixing and caused heterogeneous distribution of the viral target. Thus, higher nasal volumes allowed for sampling more viral particles from the inhomogeneous sample yielding better results. For a 12.5% sample, we sampled only 2 μL of spiked nasal fluid in a 16 μL final reaction, compared to 8 μL nasal fluid added in a 50% sample per reaction. As expected, as volume of crude nasal sample increased in the final reaction, there was a delay in amplification times as the increase in the inhibitory components causes a delay in amplification times. Hence, the amplification time increased by 3 min. for the 25% sample and by 8 min. for the 50% sample compared to the amplification times of the 12.5% sample for 5.5E5 copies/μL of viral concentration in the nasal sample. Finally, to evaluate whether sampling more of the spiked nasal fluid in a reaction improves the detection limit, we performed 96 μL reactions, where the spiked nasal fluid sample volume was 48 μL (50% nasal fluid/reaction). As shown in the supplementary information (**Figure S5**), increasing the reaction volume did not improved the LOD. Together, these results highlight that our reactions can tolerate up to 50% of crude nasal fluid samples per reaction.

3. Use of VTM

For diagnostic testing of SARS-CoV-2, the current workflow includes collecting nasopharyngeal/nasal specimens using swabs, which are immediately transferred into sterile transport tube containing 2-3 mL of VTM until diagnostic assays can be performed [34]. In standard RT-PCR assays, this VTM sample with viruses undergoes an RNA purification step next [33]. In our RT-LAMP assay, to evaluate the direct use of VTM samples without any RNA purification, we spiked serial dilutions of inactive SARS-CoV-2 viruses in VTM and performed our RT-LAMP reactions. The VTM sample was either 12.5% or 50% of the final reaction volume, and the detection limit for both reactions was 50 copies/μL (supplementary information, **Figure S6**). These results highlight no loss in the detection limit of our assay in VTM compared to SARS-CoV-2

viruses in buffer. The reactions with 50% VTM showed a delay of ~20 min. compared to the 12.5%, likely due to the increase in inhibitory components in the VTM.

4. Supply chain of resources required for standard bench-top RT-LAMP assay compared to standard RT-PCR test to perform testing at scale

We quantified the resources required to scale up the RT-LAMP assay and compare it to the conventional RT-PCR test. For each test, we considered three scenarios in which the number of patient samples are 80, 800, or 8000 (**Figure S11, Table S3, Dataset S1**).

Different scenarios require different quantities of laboratory resources. Many laboratory instruments are capable of 96 parallel tests, and thermocycler loaded with 96 tests performing the CDC RT-PCR assay would have 80 patient samples plus eight positive controls and eight negative controls [33]. Thus, we select 80 as the smallest increment of patient samples and multiples of 80 for scale up. The model accounts for the time and cost to process 16 control samples for every 80 patient samples. For the 80 patient test scenario, we assumed the use of one thermocycler, for 800 patient tests we assumed 5 thermocyclers, and for 8000 patient tests we assumed 10 thermal cyclers. Other laboratory infrastructure is required as well including refrigerated sample storage, biohazard waste management along with sufficient space to work. The cost modeling includes the costs of the reagents and disposable supplies such as swabs, pipettes, and vials, and the time averaged cost of using laboratory instruments calculated as the instrument cost divided over 10,000 hours of useful lifetime. The model does not account for the cost of space or personnel as this could vary widely. The modeling also assigns an expected time required for each step in the process. All of the costs are tracked either to the CDC test for RT-PCR [33] or the steps described in the present study for RT-LAMP.

The list of resources required shows that RT-LAMP is faster and less costly than RT-PCR, and these advantages can be linked to two key differences between the assays. First, the amplification time for RT-LAMP is about half of that for RT-PCR. RT-PCR requires time to progress through successive thermal cycles while RT-LAMP requires only one isothermal step. Second, RT-LAMP does not require a separate step for RNA extraction, saving time and reducing the cost of consumables. While the elimination of the RNA extraction kit has advantages, a disadvantage is that additional controls are required to account for the presence of the swabs. The total cost could be reduced for both techniques if the use of swabs are eliminated. For instance, if other type of specimens such as saliva could be used for the assay. Likewise, the cost of the RT-PCR technique could be potentially also reduced as recent reports show direct RT-PCR with patient nasopharyngeal [44] and saliva [5] samples without nucleic acid extraction.

Supplementary Figures

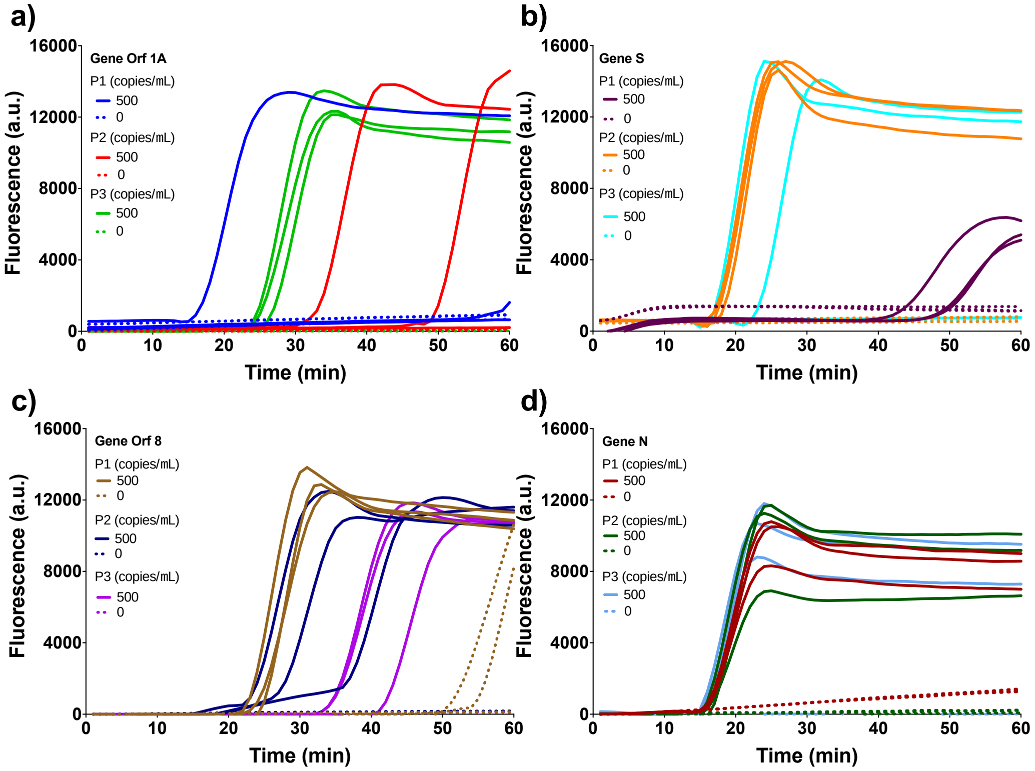

Fig. S1. Validation of 3 LAMP primer sets for 4 different SARS-CoV-2 Gene Targets. Raw fluorescence data for detecting Gene Orf 1a (***a***), Gene S (***b***), Gene Orf 8 (***c***), Gene N (***d***) of SARS-COV-2 using three different primer sets for each gene (n = 3).

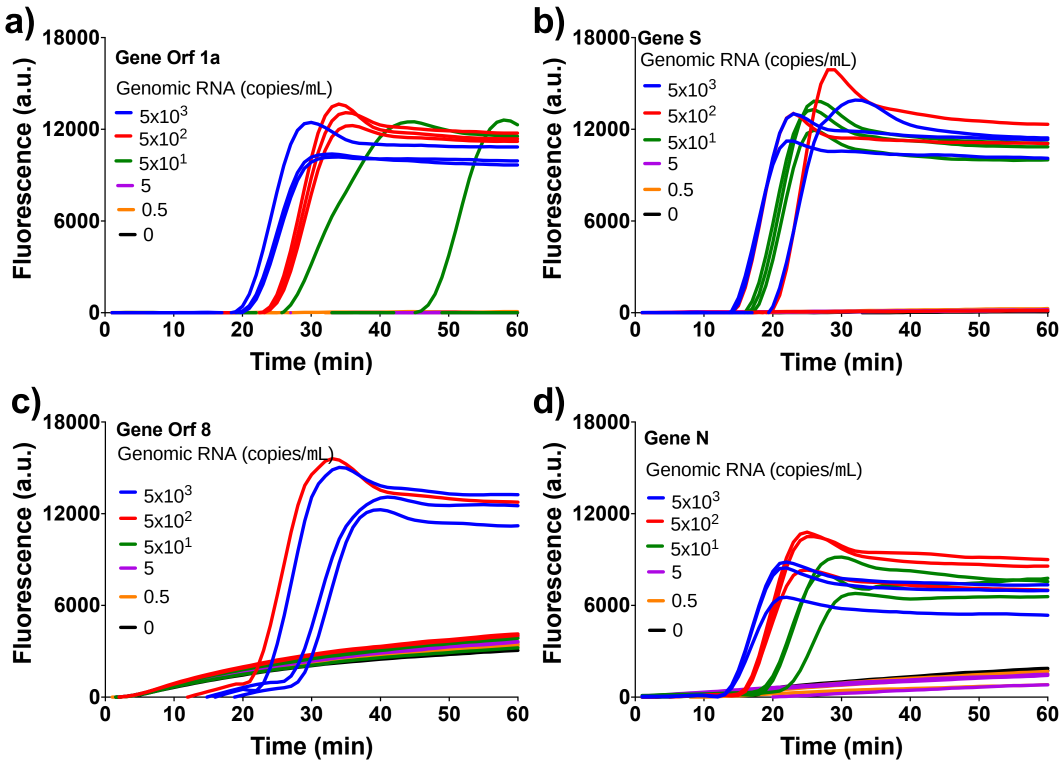

Fig. S2 Characterization of SARS-CoV-2 genomic RNA in buffer. Raw fluorescence data (n = 3) for detection of genomic RNA using ***(a)*** primer set 3 for Gene Orf 1a, ***(b)*** primer set 2 for Gene S, ***(c)*** primer set 2 for Gene Orf 8, ***(d)*** and primer set 1 for Gene N. The best detection limit was 50 copies/μL attained using Gene N primer set 1.

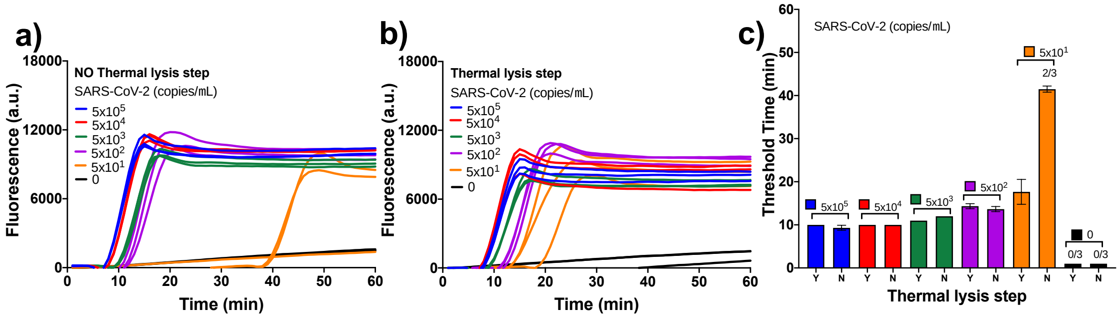

Fig. S3 Characterization of thermal lysis of inactive SARS-CoV-2 virus in buffer. (*a-b*) Raw fluorescence data and ***(c)*** amplification threshold times (n = 3) for inactive SARS-CoV-2 virus detection without and with thermal lysis (95°C, 1 min) step prior to the final reaction. The bar graphs show mean and standard deviation. Y = Yes. N = No.

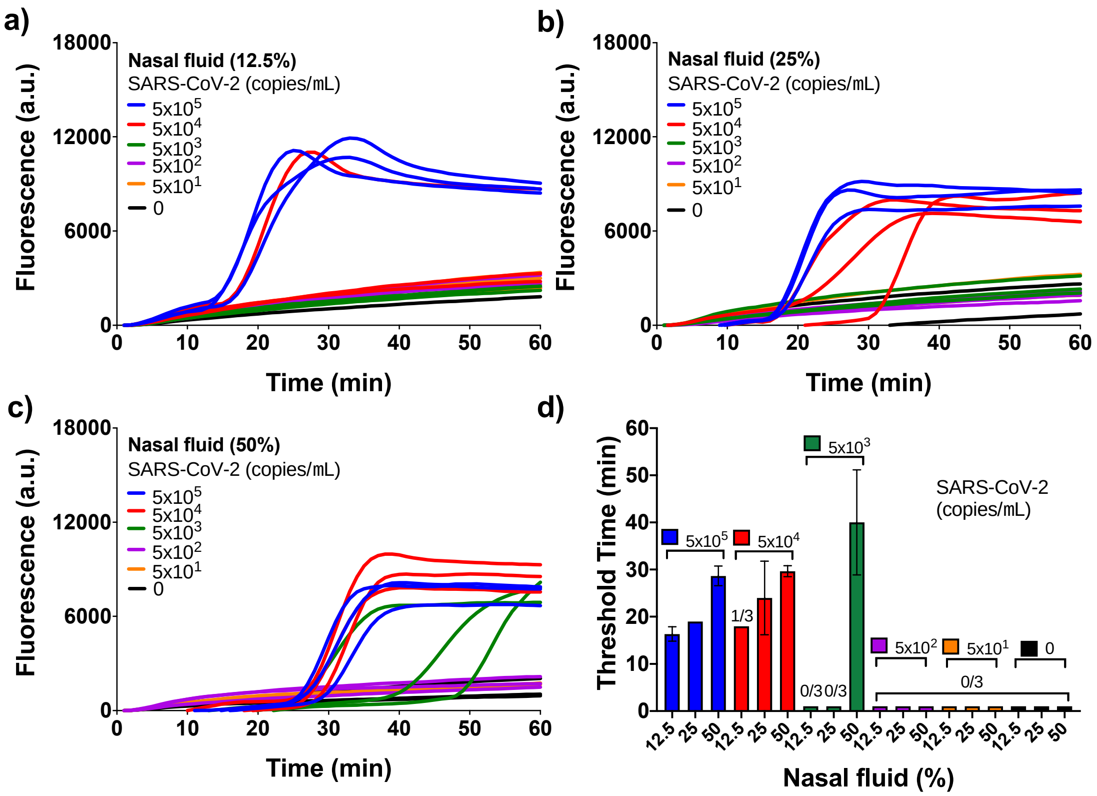

Fig. S4. Characterization of SARS-CoV-2 virus in nasal fluid in a 16 μL reaction. (*a-c*) Raw fluorescence data and ***(d)*** amplification threshold times (n = 3) for viral detection in a 16 μL reaction with 12.5%**,** 25%*,* and 50% spiked nasal fluid per reaction. The bar graphs show mean and standard deviation.

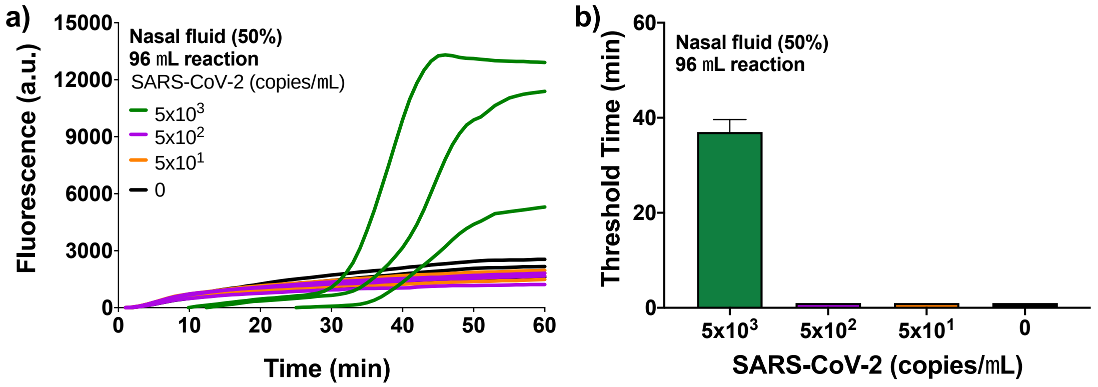

Fig. S5. Characterization of SARS-CoV-2 virus in nasal fluid in a 96 μL reaction. (*a-b*) Raw fluorescence data and ***(c)*** amplification threshold times (n = 3) for viral detection in a 96 μL reaction with 50% nasal fluid per reaction. Thermal lysis at 95°C was conducted for 1 min. of the virus in nasal fluid sample before addition of RT-LAMP reagents for the final reaction. The bar graphs show mean and standard deviation. Fraction indicates number of replicates amplified.

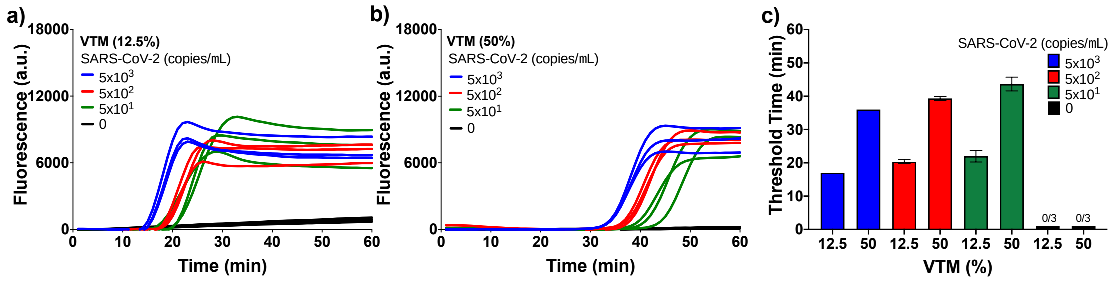

Fig. S6. Characterization of SARS-CoV-2 virus in VTM. (*a*-*b*) Raw fluorescence data and ***(c)*** amplification threshold times (n = 3) for viral detection in a 16 μL reaction with **(*a*)** 12.5% and **(*b*)** 50% spiked VTM per reaction. Thermal lysis at 95°C was conducted for 1 min. of the virus in VTM sample before addition of RT-LAMP reagents for the final reaction. The bar graphs show mean and standard deviation.

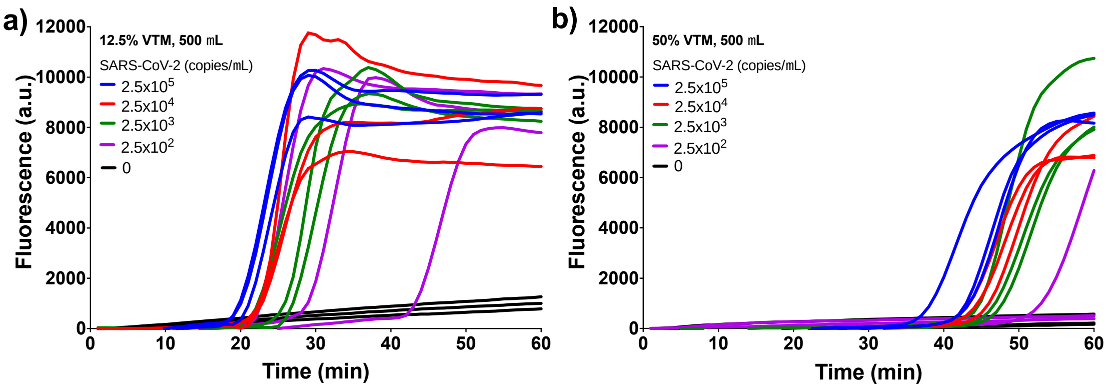

Fig. S7 Characterization of SARS-CoV-2 virus in mock nasopharyngeal swab samples transported in 500 μL of VTM. Raw fluorescence data (n = 3) for viral detection in a 16 μL reaction with **(*a*)** 12.5% and **(*b*)** 50% VTM sample per reaction from a 500 μL VTM sample.

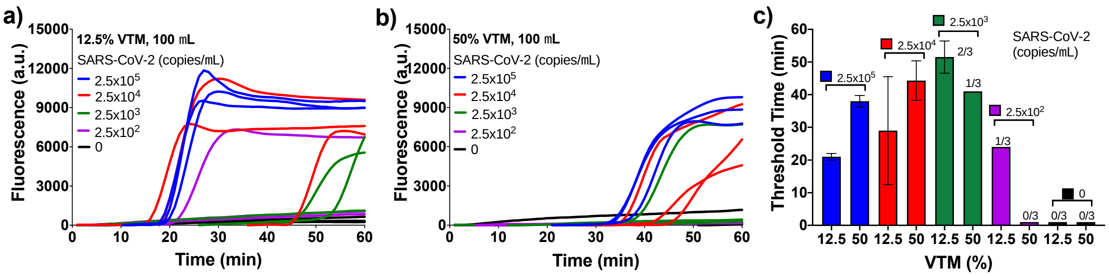

Fig. S8. Characterization of SARS-CoV-2 virus in mock swab samples transported in 100 μL of VTM. (***a-b***) Raw fluorescence data and ***(c)*** amplification threshold times (n = 3) for viral detection in a 16 μL reaction with 12.5% and 50% VTM per reaction from a 100 μL VTM sample. The bar graphs show mean and standard deviation.

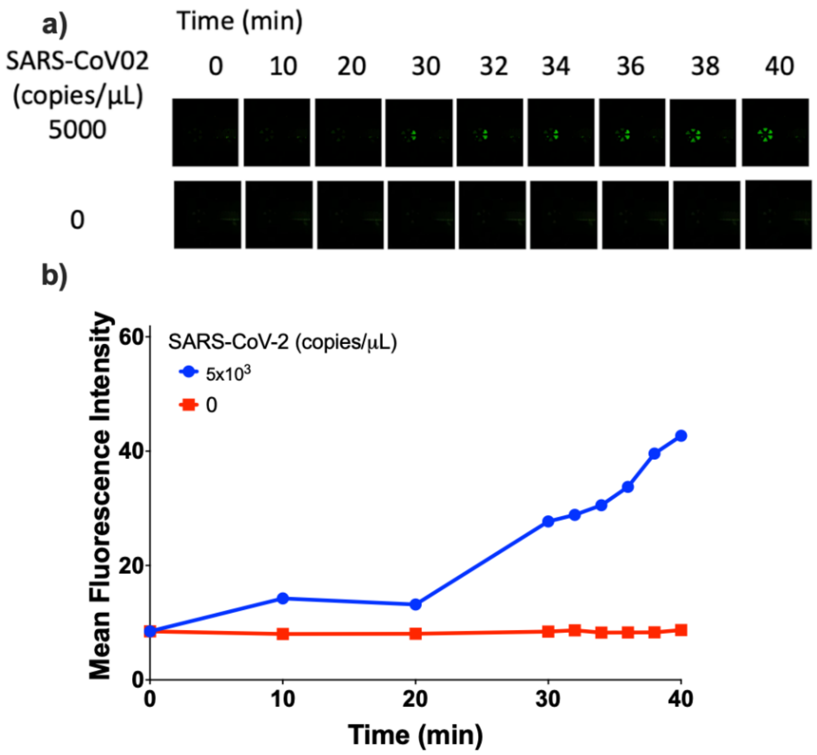

Fig. S9. Rapid Detection of SARS-CoV-2 in spiked VTM and using an additively manufactured cartridge and handheld point of care instrument. (*a*) Baseline-subtracted fluorescence images of real-time RT-LAMP on the additively manufactured amplification chip at different time points showing the amplification of 5000 and 0 copies/μL of inactivated SARS-CoV- 2 virus in VTM. **(*b*)** Baseline subtracted mean fluorescent intensity over time for on-chip amplification detection.

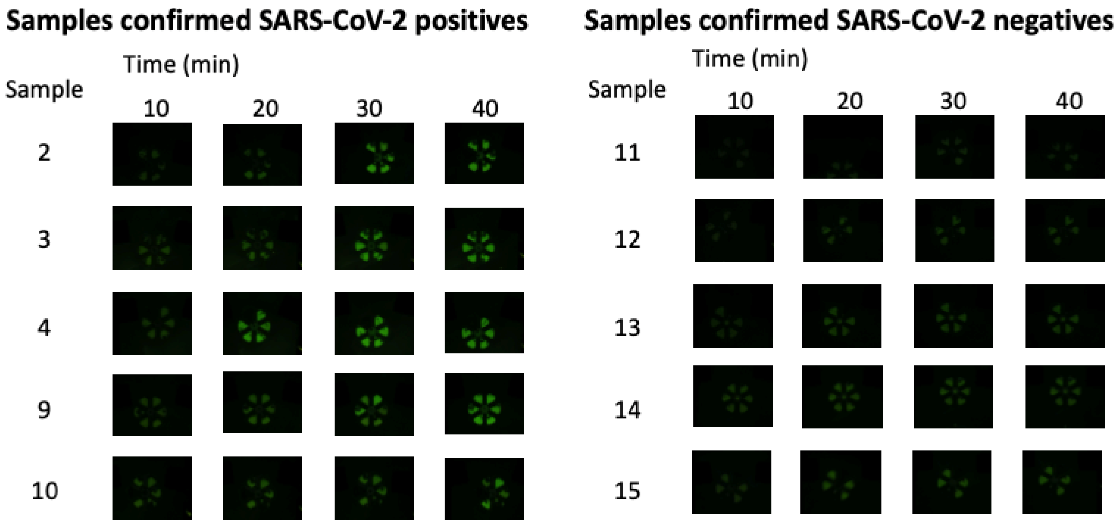

Fig. S10. Rapid Detection of SARS-CoV-2 in VTM clinical samples using an additively manufactured cartridge and handheld point of care instrument. (*a*) Fluorescence images of real-time RT-LAMP SARS-CoV-2 analysis on the additively manufactured amplification chip at different time points.

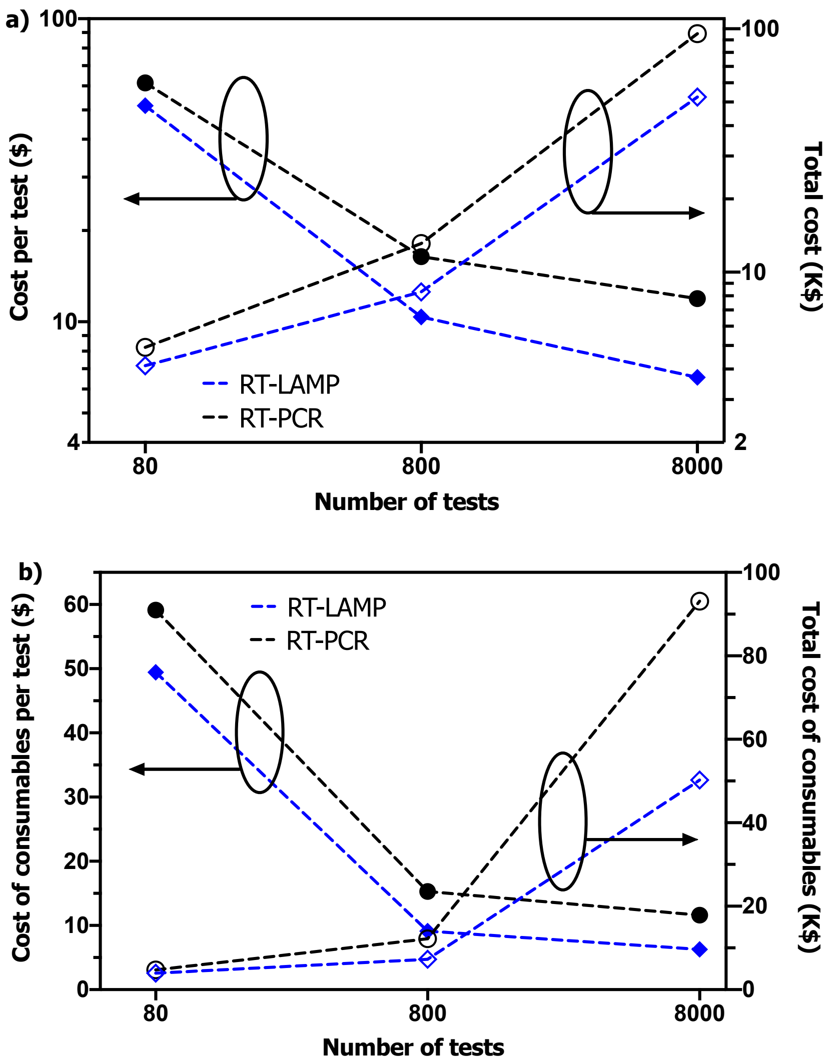

Fig. S11. Resources modeling results. (*a*) Cost per test and total cost for the three scenarios considered. **(*b*)** Total cost of consumables and Cost of consumables per test for the 3 scenarios considered. (Approximate estimates)

Supplementary Tables

Table S1. RT-LAMP primer sequences for 12 primer sets (3 primer sets each for 4 target genes)

**Gene Orf1a**	**P1**	F3	ATCTAGTTGTAATGGCCTACA
		B3	ACAAGCACAGGTTGAGAT
		FIP	TGAGTTTTTCATAAACAGTGCCAAA-CAGGTGGTGTTGTTCAGT
		BIP	AACCCGTCCTTGATTGGCTT-AATTTAACAATTTCCCAACCGTC
		Loop F	TGTTAGTTAGCCACTGCGAAGT
	**P2**	F3	AAACCCGTCCTTGATTGG
		B3	CTTTAAGTTTAGCTCCACCAAT
		FIP	TCACAAGCACAGGTTGAGATAAATT-GAAGGTGTAGAGTTTCTTAGAGAC
		BIP	GTCACCTGTGCAAAGGAAATTAAG-AGAGTCAGCACACAAAGC
	**P3**	F3	CGGTGGACAAATTGTCAC
		B3	CTTCTCTGGATTTAACACACTT
		FIP	TCAGCACACAAAGCCAAAAATTTAT-CTGTGCAAAGGAAATTAAGGAG
		BIP	TATTGGTGGAGCTAAACTTAAAGCC-CTGTACAATCCCTTTGAGTG
**Gene S**	**P1**	F3	TCTTTCACACGTGGTGTT
		B3	CAGTGGAAGCAAAATAAACAC
		FIP	GAAAGGTAAGAACAAGTCCTGAGT-TTACCCTGACAAAGTTTTCAG
		BIP	TTCCAATGTTACTTGGTTCCATGC-GACAGGGTTATCAAACCTCT
		Loop B	TATACATGTCTCTGGGACCAATGG
	**P2**	F3	GGTGTTTATTACCCTGACAAAG
		B3	GTACCAAAAATCCAGCCTC
		FIP	TGGAACCAAGTAACATTGGAAAAGA-TTTTCAGATCCTCAGTTTTACATTC
		BIP	CTCTGGGACCAATGGTACTAAGAG-GACTTCTCAGTGGAAGCA
		Loop B	AACCCTGTCCTACCATTTAATGATG
	**P3**	F3	CCTGACAAAGTTTTCAGATCC
		B3	GTACCAAAAATCCAGCCTC
		FIP	GCATGGAACCAAGTAACATTGGAAA-TCAGTTTTACATTCAACTCAGGA

		BIP	CTCTGGGACCAATGGTACTAAGAG-GACTTCTCAGTGGAAGCA
		Loop B	AACCCTGTCCTACCATTTAATGATG
**Gene Orf 8**	**P1**	F3	ACGCCTAAACGAACATGAA
		B3	AGAACCAGCCTCATCCAG
		FIP	GGTTGATGTTGAGTACATGACTGTA-CTTGTTTTCTTAGGAATCATCACA
		BIP	ATATGTAGTTGATGACCCGTGTCC-TAAAGGTGCTGATTTTCTAGCT
		Loop F	CTACATTCTTGGTGAAATGCAGCTA
	**P2**	F3	CTCAACATCAACCATATGTAGT
		B3	CAATTTAGGTTCCTGGCAATT
		FIP	GGTGCTGATTTTCTAGCTCCTACTC-GACCCGTGTCCTATTCAC
		BIP	TGCTGGATGAGGCTGGTTCTA-TGTAAAAGGTAACAGGAAACTG
		Loop B	ATCACCCATTCAGTACATCGATATC
	**P3**	F3	AGCTGCATTTCACCAAGAA
		B3	CGATATCGATGTACTGAATGG
		FIP	TGAATAGGACACGGGTCATCA-GTAGTTTACAGTCATGTACTCAA
		BIP	GAGTAGGAGCTAGAAAATCAGCAC-TGATTTAGAACCAGCCTCATC
**Gene N**	**P1**	F3	GTTCCTCATCACGTAGTCG
		B3	GTTTGGCCTTGTTGTTGTT
		FIP	GCCAGCCATTCTAGCAGGAG-CAACAGTTAAGAAATTCAACTCC
		BIP	GATGCTGCTCTTGCTTTGCT-ACCAGACATTTTGCTCTCAA
		Loop B	GCTGCTTGACAGATTGAACCAG
	**P2**	F3	AGACGAATTCGTGGTGGT
		B3	TTGTTAGCAGGATTGCGG
		FIP	TGGCCCAGTTCCTAGGTAGT-GACGGTAAAATGAAAGATCTCAG
		BIP	CTTCCCTATGGTGCTAACAAAGAC-TGGTGTATTCAAGGCTCC
		Loop B	GGCATCATATGGGTTGCAACTGAG
	**P3**	F3	GTCATTTTGCTGAATAAGCATAT
		B3	GAGTCAGCACTGCTCATG

		FIP	TAAGGCTTGAGTTTCATCAGCCTT-ACGCATACAAAACATTCCCA
		BIP	CAGAGACAGAAGAAACAGCAAACT-GATTGTTGCAATTGTTTGGAG
		Loop B	GTGACTCTTCTTCCTGCTGCAGATT

Table S2. Threshold times (off-chip RT-LAMP assay) obtained after analysis of the confirmed positive samples (RT-PCR control).

Sample ID	RT-LAMP assay (threshold time, n = 4)			
1	19	20	19	19
2	23	24	23	22
3	24	25	25	24
4	18	18	18	18
5	21	21	21	21
6	22	22	22	22
7	22	22	22	21
8	16	17	17	16
9	42	20		19
10	24	24	24	24

Table S3. Cost and personnel required to perform the analysis of 80, 800, and 8000 samples in bench top RT-PCR versus RT-LAMP (Approximate estimates)

**Number of tests**	**80**		**800**		**8000**	
**Technique**	RT-LAMP	RT-PCR	RT-LAMP	RT-PCR	RT-LAMP	RT-PCR
**Cost per test excluding labor ($)**	51.7	61.4	10.4	16.4	6.6	11.9
**Consumables cost per test ($)**	49.4	59.1	9.1	15.3	6.3	11.6
**Total time (h)**	5.5	6.9	10.9	13.8	54.6	68.9
**Number of thermocyclers assumed**	1		5		10	

Dataset S1. Supply chain: RT-LAMP and RT-PCR assay

See excel file.

Supplementary video

Video S1. Uniform filling of the amplification chambers, time stamped video of amplification on the cartridge for 5000 copies/μL of virus in VTM and negative control (VTM only).

See video
